# Genetic diversity of Plasmodium Vivax in South of Iran: A cross-sectional study

**Published:** 2015

**Authors:** K Sharifi-Sarasiabi, S Hosseiniteshnizi, F Dehghan, A Madani

**Affiliations:** * Molecular Medicine Research Center, Hormozgan University of Medical Sciences, Bandar Abbas, Iran and Khalij Fars Hospital, Bandar Abbas, Iran; ** Department of Biostatistics, Faculty of Para-Medicine, Hormozgan University of Medical Sciences, Bandar Abbas, Iran; *** Infectious and Tropical Diseases Research Center, Hormozgan University of Medical Sciences, Bandar Abbas, Iran; **** Social Determinants in Health Promotion Research Center, Hormozgan University of Medical Sciences, Bandar Abbas, Iran

**Keywords:** Plasmodium vivax, malaria, protozoan circumsporozoite protein, malaria vaccines, Iran

## Abstract

Despite declining the number of malaria cases in Iran, increased prevalence of malaria is supposed to be due to migration from eastern neighboring countries of Iran, which are abundant in Plasmodium vivax (P. vivax). The circumsporozoite protein (CSP) of the P. vivax, is one of the candidate antigens for antimalaria vaccine. The diversity of P. vivax populations circulating in Iran has been investigated by using circumsporozoite protein (CSP) in this study. A hundred and eighteen blood samples were collected from patients diagnosed with P. vivax malaria from south of Iran during 2007-2008. All samples were analyzed by using nested PCR/ RFLP and 18 were sequenced. Genotyping of Pvcsp gene showed that VK210 type was predominant (95%) in south of Iran. Sequence analysis of Pvcsp gene revealed 6 distinct allelic variants in VK210 type. The present data indicate that there is some degree of genetic diversity among P. vivax populations in Hormozgan province of Iran. It seems that in neighbors of Iran, VK210 type is predominant, probably due to similar vector of malaria in these regions.

## Introduction

Among the human malaria parasites, Plasmodium vivax is the most geographically widespread species in the tropical and subtropical regions of the word [**1**, **2**].P vivax remains a major obstacle in controlling malaria [**3**] in Iran, located in the Eastern Mediterranean region in central Asia [**4**]. In 2011, centers for disease control and prevention (CDC) reported that the number of malaria cases has decreased to 2656 cases [**5**] and P. vivax was the cause of approximately 90% of all malaria infections[**6**] Despite the low incidence of malaria [**7**] , its high prevalence in Iran could be related to the migration of people from neighboring countries (i.e. Pakistan and Afghanistan). This can be interpreted by taking a look at the annual malaria cases in Pakistan and Afghanistan which are 500000 cases and 3 million cases respectively [**8**]. Due to the fact that P. vivax is the predominant plasmodium species in these countries, research on intervention strategies to control this parasite appeared to be essential [**9**, **10**]. Designing effective vaccines is one of the control strategies, but because of genetic diversity in natural parasite populations, the development of an effective vaccine has been limited [**11**]. Even though prevention, diagnosis, and treatment of P. vivax malaria is difficult, most efforts in eliminating malaria and action of malaria vaccines are focused on P. falciparum [**12**]

The circumsporozoite protein (CSP) of the P. vivax which, is the most abundant protein on the surface sporozoite, is one of the candidate antigens for antimalarial vaccine [**13**-**15**]. Based on sequence analyses, Pvcsp consists of three different alleles, VK210, VK247 and P. vivax-like with one of three types of nonapeptide repeat units GDRA(A/D)GQPA, ANGA(G/D)(N/D)QPG and APGANQ(E/G)GGAA respectively [**16**-**18**]. CSP variant (VK210, VK247/P.vivax-like) was found in clinical isolates of P. vivax, thus CSP serves as a helpful tool for genotyping [ **18**- **20**].

The Ministry of Health and Medical Education of Iran initiated a national malaria elimination program with the goal of eliminating malaria by 2025, and molecular studies on circulating species of P. vivax in endemic area in Iran will provide information to achieve this important goal. Therefore, this study’s aim was to detect the genetic diversity in CSP gene of P. vivax in one of the endemic areas of malaria in Iran. The genetic diversity of CSP is distinguished with PCR-RFLP technique & DNA sequencing, which may assist future management of P. vivax malaria and designing an effective malaria vaccine.

## Materials & Methods 

**Fig. 1:**
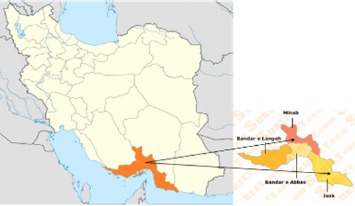
Map of Iran showing the location of the study region. http://www.safareiran.com

A total of 118 blood samples were taken from P. vivax infected patients after an initial diagnosis by Giemsa-stained thin and thick blood smears. These samples were collected in 2007 and 2008 from two malaria endemic areas of Hormozgan province including Minab (46 samples) and Bandar-e-Jask (72 samples) in the south of Iran (**Fig. 1**)

**Genomic DNA extraction & PCR analysis for *Pvcsp* gene**

Genomic DNA was extracted from 100μl of each blood sample with the DNG PLUS extraction kit (Cinnagen, Iran) according to the manufacturer's recommendations. The quality of the isolated DNA from each specimen was determined by electrophoresis on 1% agarose gel containing ethidium bromide and visualized by an UV transilluminator. DNA samples were stored at -20°C until PCR was performed.

A nested PCR method was implemented to amplify the repeat regions of *Pvcsp* gene by using 2 sets of primers as described by Henry-Halldin et al [**2**] The oligonucleotide primers and expected PCR product with the appropriate size are shown in **Table 1**

**Table 1:** Primers sequence in this study

Primer names	Primer sequence	size of PCR product
*PVCS1F*	ATGTAGATCTGTCCAAGGCCATAAA	1100bp
*PVCS1R*	TAATTGAATAATGCTAGGACTAACAATAG	
*PVCS2F*	GCAGAACCAAAAAATCCACGTGAAAATAAG	680bp
*PVCS2R*	CCAACGGTAGCTCTAACTTTATCTAGGTAT	

The first PCR reaction was performed in a total volume of 25μl containing: 1μl DNA template, 1μl of each primer at 10 pmol/ μl, 0.5μl of deoxyribonucleotide triphosphate (dNTP) (200mM), 2μl buffer 10X (100mM Tris-Hcl (pH 8.8), 500 Mm Kcl), 1.5μl mgcl2 and 1 unit Tag DNA polymerase (Cinnagen, Iran).

PCR conditions consisted of an initial denaturation at 95°C for 5 min followed by 30 cycles of denaturation at 95°C for 1 min, annealing at 58°C for 1 min and extension at 72°C for 1 min with a final extension at 72°C for 10 min

The second PCR reaction was performed in a total volume of 50μl containing: 3μl of 1:50 diluted of first PCR product in distilled water, 2μl of each primer, 1μl of dNTP (200mM), 4μl buffer 10x, 2.5μl mgcl2 and 2U Tag DNA polymerase. The PCR conditions were the same as the first PCR except for the fact that the annealing temperature was increased to 62°C. PCR amplified products were vitalized by gel Doc system after electrophoresis on 2% agarose gel.

**Genotyping *Pvcsp* gene by PCR–RFLP**

The repeat regions of the Pvcsp gene for the two major types, VK210 and VK247 were genotyped by PCR–RFLP, as previously described [**25**]. To distinguish the two types of P. vivax repeats, VK210 and VK247, the PCR products were separately digested with Alu I or BstN1 (MBI, Fermentase, Lithuania) in a total volume of 20 μl for 3 hrs according to the supplier’s instructions. Alu I sites were not present in VK247 sequence, while those of VK210 harbors and BstN1 sites were not seen in VK210 sequence, unlike the numerous sites in VK247 sequence[**23**- **25**]

The electrophoresed DNA fragments were visualized on an ultraviolet transilluminator following ethidium bromide staining by electrophoresis on 2% agarose gel.

**Sequencing**

DNA sequencing analysis was performed in both directions for 18 PCR products, which had 6 different restriction patterns in an ABI 3130 Genetic analyzer with a BigDye Terminator V3.1 cycle sequencing kit. The sequences were aligned by using Gene Runner Software (Version 3.05) and then compared with previously sequences of Pvcsp gene available in GenBank with the MULT align program.

## Results

**Fig. 2:**
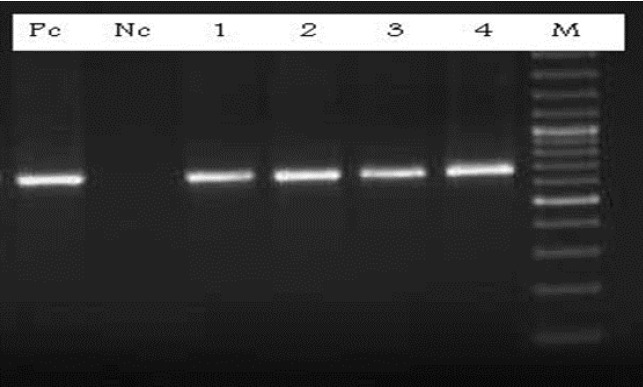
Nested-PCR product of Pvcsp gene. Pc=Positive control, Nc= negative control, 1-4= samples, M= Marker (100bp)

The participants of the study were 79 male (67%) and 39 female (33%) patients aged 470 years. The result of DNA amplification by the nested PCR method based on the primers used in this study showed the same size of approximately 680 bp for the Pvcsp gene in the DNA preparation obtained from all isolates (**Fig 2**).

Among all samples, VK210 genotype was observed in 95% of P. vivax parasites and 5% had a mixed genotype (VK210 and VK247), while isolated VK247 genotype was absent. Six different banding patterns were revealed in VK210 genotype (**Fig 3a**,**b**) and from each pattern three samples were sequenced. The nucleotide sequences data reported in this article have been submitted to GenBank databases under the accession numbers KM496318-KM496322 and KM575832.

**Fig. 3a:**
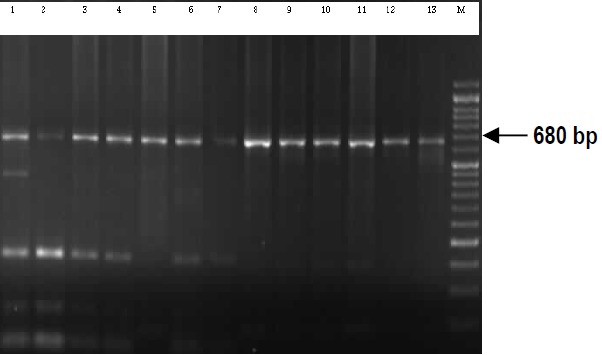
PCR-RFLP analysis of *Pvcsp* gene by BstN1 restriction enzyme. M: Marker (50bp), Mixed VK210 & VK247: 1, 2, 3, 4, 6,7 and VK210: 5, 8, 9, 10, 11, 12, 13

**Fig. 3b:**
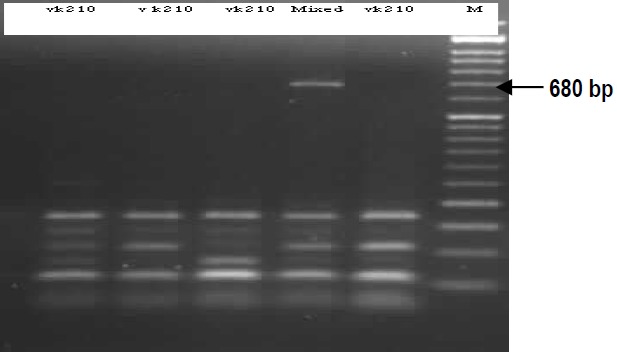
PCR-RFLP analysis of *Pvcsp* gene by Alu1 restriction. M: Marker (50bp), Mixed: VK210 and VK247

## Discussion

Countrywide malaria control programs starting in 1958 in Iran led to an important reduction of malaria infection in three southeastern and southern provinces including Sistan & Baluchistan, Hormozgan and tropical region of Kerman. However, P. vivax was the main causative malaria infections in these areas [**4**].

The analysis of the genetic diversity of P. vivax could assist in distinguishing different genotypes present in the population and choose the appropriate approach for the management of malaria [**21**].

In the present study, genotyping of Pvcsp gene was analyzed by using the PCR-RFLP method by two restriction enzyme Alu1 % BstN1. These enzymes were chosen according to the PCR product size. Alu1 was not adequate to identify VK247 in mixed infections with the P. vivax-like genotype. We solved this problem by adding BstN1 that allowed us to separate the VK247 and P. vivax-like genotype in the mixed infection [**22**].

The genotyping of Pvcsp gene revealed that VK210 was the most prevalent genotype in isolates from these areas. This finding is consistent with results from studies carried out in Iran (70.5%), Pakistan (85.5%), Guyana (92%), Brazil (86%), Afghanistan (87.4%), India (99.3%) and Thailand (90%) [**10**,**19**,**23**-**27**].

In contrast with previous studies [**14**,**23**,**28**], VK247 genotype was not isolated from any samples in this study. No P. vivax-like parasites were detected in our study, however, parasite containing this nonapeptide was found in isolates from Brazil, Madagascar, and Indonesia [**10**].

In addition, mixed genotype infection was scant, which was supported by finding of Zakeri et al. in Pakistan (1.7%) [**29**], in Afghanistan (6.4%) [**23**], in southeast of Iran (11.7%) [**10**] and Bonilla in Guyana (9%) [**26**].

These discrepancies seem to have arisen from the susceptibility of various vector species to the infections by different parasites types, sample size biases, geographical region, and rate of population movement [**4**,**19**,**30**,**31**].

Because PCR RFLP technique had limitations in genotyping of malaria parasite such as being dependent on size of restriction fragments % sensitivity of chosen PCR reaction [**32**], also, sequence analysis provided more information about genetic polymorphisms of the P. vivax samples [**9**], thus, the sequencing of PCR product of Pvcsp gene was carried out and illustrated that there were 5 new genetic variations and polymorphisms in these isolates. Two synonymous substitutions were based on amino acid sequences and therefore 3 polymorphisms were observed.

The limitations of this study were the following: first, the sequencing was performed for limited numbers of samples, thus probably by increasing the number of samples for sequencing; more genetic polymorphisms in Pvcsp gene could be detected. Second, it did not include all of the P. vivax isolates which were circulating among patients of the mentioned regions in south of Iran.

## Conclusion

Although few samples were sequenced in this study, and the data may not represent the whole picture of genome diversity of the parasite population in the region, the results suggested parasite populations with relatively diverse genetic backgrounds in the south of Iran. VK210 is predominant in eastern neighbors of Iran, which could be probably due to similarity of vectors and could be used for designing a DNA vaccine for malaria.

**Acknowledgements**

Authors would like to acknowledge Mohseni GH, Taghizadeh N, Nejadeydi M, Ahmadpour H, Yerian M, Rasti H, and Ameri K, in Hormozgan University of Medical Sciences and Health Services, for their useful contributions in the study.

**Conflict of interest**

Authors declare they have no conflict interest.

**Funding organization**

Molecular Medicine Research Center, Hormozgan University of Medical Sciences.
